# Application of Bio-Friendly Formulations of Chitinase-Producing *Streptomyces cellulosae* Actino 48 for Controlling Peanut Soil-Borne Diseases Caused by *Sclerotium rolfsii*

**DOI:** 10.3390/jof7030167

**Published:** 2021-02-25

**Authors:** Gaber Abo-Zaid, Ahmed Abdelkhalek, Saleh Matar, Mai Darwish, Muhammad Abdel-Gayed

**Affiliations:** 1 Bioprocess Development Department, Genetic Engineering and Biotechnology Research Institute (GEBRI), City of Scientific Research and Technological Applications (SRTA-City), New Borg El-Arab City, Alexandria 21934, Egypt; gaberam57@yahoo.com (G.A.-Z.); salehmatar@yahoo.com (S.M.); 2 Plant Protection and Biomolecular Diagnosis Department, ALCRI, City of Scientific Research and Technological Applications, New Borg El Arab City, Alexandria 21934, Egypt; 3 Chemical Engineering Department, Faculty of Engineering, Jazan University, Jazan 45142, Saudi Arabia; 4 Botany and Microbiology Department, Faculty of Science, Alexandria University, Alexandria 21526, Egypt; maidarwish33@gmail.com; 5 Onion, Garlic and Oil Crops Diseases Research Department, Plant Pathology Research Institute, Agricultural Research Center, Giza 12619, Egypt; ma.abdelgayed@yahoo.com

**Keywords:** peanut, *Sclerotium rolfsii*, *Streptomyces cellulosae*, chitinase, bio-friendly formulations, biocontrol, qRT-PCR

## Abstract

Of ten actinobacterial isolates, *Streptomyces cellulosae* Actino 48 exhibited the strongest suppression of *Sclerotium rolfsii* mycelium growth and the highest chitinase enzyme production (49.2 U L^−1^ min^−1^). The interaction between Actino 48 and *S*. *rolfsii* was studied by scanning electron microscope (SEM), which revealed many abnormalities, malformations, and injuries of the hypha, with large loss of *S*. *rolfsii* mycelia density and mass. Three talc-based formulations with culture broth, cell-free supernatant, and cell pellet suspension of chitinase-producing Actino 48 were characterized using SEM, Fourier transform infrared spectroscopy (FTIR), and a particle size analyzer. All formulations were evaluated as biocontrol agents for reducing damping-off, root rot, and pods rot diseases of peanut caused by *S*. *rolfsii* under greenhouse and open-field conditions. The talc-based culture broth formulation was the most effective soil treatment, which decreased the percentage of peanut diseases under greenhouse and open-field conditions during two successive seasons. The culture broth formulation showed the highest increase in the dry weight of peanut shoots, root systems, and yielded pods. The transcriptional levels of three defense-related genes (*PR-1*, *PR-3*, and *POD*) were elevated in the culture broth formulation treatment compared with other formulations. Subsequently, the bio-friendly talc-based culture broth formulation of chitinase-producing Actino 48 could potentially be used as a biocontrol agent for controlling peanut soil-borne diseases caused by *S*. *rolfsii*.

## 1. Introduction

Peanut, or groundnut (*Arachis hypogaea* L.), is one of the most important oilseed crops in the world, including Egypt. Peanut is susceptible to diseases caused by abundant soil-borne pathogens. One of the most important soil-borne fungal diseases of peanut is stem, root, and pods rot caused by *Sclerotium rolfsii* (teleomorph *Athelia rolfsii* (Curzi) C.C. Tu & Kimbr.), which has the ability to infect more than 500 plant species [1]. Circular and light-tan to brown clusters of seed-like bodies less than 1/10th of an inch in diameter called sclerotia form on the mat of fungal growth on the soil surface, decaying stems and pods and other crop debris. These sclerotia enable the fungus to survive for extensive periods on plant debris and the soil [2]. Chet [3] reported that the presence of melanin in the rind cells of the sclerotia may be responsible for their ability to survive for long durations and avoid degradation by chemicals and microorganisms. Consequently, the management of *S*. *rolfsii* is difficult.

Applying fungicides for controlling soil-borne fungi has many disadvantages such as environmental pollution, pathogen resistance, and the hazards posed to human and animal health [4,5]. Additionally, imbalances in the microbial community may be caused by the extreme use of fungicides [5]. Biocontrol agents are gradually being substituted for pesticides for controlling plant diseases since biological control is an environmentally friendly approach and safe for humans and animals [4,6].

*Streptomyces* species are Gram-positive and spore-producing bacteria with filamentous growth and a higher G≡C content of more than 70%. A variety of active compounds for agricultural applications are produced using *Streptomyces* spp. [7]. Various species of *Streptomyces* have effects in the biocontrol of soil-borne phytopathogenic fungi such as *Fusarium oxysporum*, *Pythium ultimum*, *Rhizoctonia solani*, and *S*. *rolfsii*. Biocontrol of these fungi can be achieved by diverse mechanisms such as the production of antibiotics and siderophores, hyper-parasitism, and induction of the plant resistance response [8,9,10,11,12]. Intra et al. [13] suggested that rhizospheric soil is an attractive source for the discovery of actinomycetes with activity against *Colletotrichum* spp. A strain (JF-1) with high inhibitory activity has the potential to produce a new compound that may be useful in the control of *Colletotrichum* spp. *Streptomyces* spp. are able to produce various lytic enzymes, such as chitinases and cellulases, which allow it to degrade and grow on insoluble organic polymers, like chitin and cellulose [14]. Gupta et al. [15] reported that actinobacteria are one of the main groups of chitinolytic microorganisms. Chitinases production by actinobacteria, especially *Streptomyces* spp., plays a vital role for these organisms in the biocontrol efficacy of phytopathogenic fungi. The production of chitinases by *Streptomyces* spp. supports their capability to obtain nutrients during degradation of environmental chitin, including that found in the cell wall of soil fungi. For that reason, *Streptomyces* spp. can be used as a biocontrol agent for the management of plant pathogenic fungi [14]. *Streptomyces* spp. play an important role in promoting plant growth due to their ability to secret some metabolites known as hormones, which promote and improve plant growth [16]. *S*. *atrovirens* isolated from groundnut roots showed promoting activity on groundnut and a number of other crops [17]. *S*. *mutabilis*, *S*. *atroolivaceus,* and *S*. *filipinensis* efficiently promote plant growth as a result of their production of indole-3-acetic acid (IAA) and 1-aminocyclopropane-1-carboxylate (ACC) [18,19,20]. These results support the use of *Streptomyces* as plant growth stimulants. 

Some biofungicides based on *Streptomyces* species were developed and commercialized, e.g., Actinovate depends on *S*. *lydicus*, Mycostop is derived from *S*. *griseoviridis*, and Rhizovit contains *Streptomyces* sp. DSMZ 12424 [21,22,23]. Consequently, *Streptomyces* species represent an important source of biofungicides and biofertilizers for agricultural application. Boukaew et al. [24] used a bioformulation of *S*. *philanthi* RL-1-178 to control root and stem rot of chili pepper caused by *S. rolfsii*. Jacob et al. [25] reported that talc-based formulation of *Streptomyces* sp. RP1A-12 (45%) decreased the disease incidence of peanut stem rot caused by *S*. *rolfsii*.

The main objective of this study was to evaluate the antagonistic effect of some actinobacterial isolates against *S*. *rolfsii*, the causal agent of peanut root and pods rot disease. We aimed to evaluate efficiency of talc-based culture broth, cell-free supernatant, and cell pellet formulations of chitinase-producing *S*. *cellulosae* Actino 48 for reducing damping-off, root rot, and pods rot of peanut under greenhouse and open-field conditions. We also studied the transcriptional changes of three defense-related genes (pathogenesis related-protein 1 (*PR-1*), chitinase (*PR-3*), and peroxidase (*POD*)) using quantitative realtime-polymerase chain reaction (qRT-PCR) in the response to *S*. *rolfsii* infection and treatment with bio-formulations.

## 2. Materials and Methods

### 2.1. Fungal and Actinobacterial Isolates

The *S*. *rolfsii* isolate used in this study was previously provided by Dr. Muhammad A. Abdel-Gayed, Onion, Garlic and Oil Crops Research Department, Plant Pathology Research Institute, Agricultural Research Center (Giza, Egypt), and its pathogenic capability was determined, consequently.

Ten actinobacterial isolates were provided by Dr. Gaber A. Abo-Zaid, City of Scientific Research and Technological Applications (SRTA-City) (Alexandria, Egypt). Depending on the 16S rDNA sequence, Actino 48 was identified as *S*. *cellulosae* and deposited in GenBank under accession number MT573878.

### 2.2. Antagonistic Effect of Actinobacterial Isolates against S. rolfsii

Estimation of *S*. *rolfsii* biomass development in the presence of actinobacterial isolates, to determine their antagonistic effect, was performed according to Trivedi et al. [26] with slight modifications. We added 1 mL of the 5-day-old pre-culture of actinobacterial isolates into 50 mL potato dextrose broth (PDB) containing *S*. *rolfsii* plug (6 mm diameter) from freshly grown culture on potato dextrose agar (PDA). The media were incubated at 30 °C for seven days. Another flask containing PDB with only the fungal plug was used as a control. The contents of the flasks were filtered through pre-weighed Whatman No. 1 filter paper and allowed to dry at 50 °C. The percentage of weight reduction of the tested fungus was calculated using the formula: (W1 − W2)/W1 × 100, where W1 represents the weight (g) of the tested fungus in a control flask and W2 is the weight of the fungus in the presence of antagonistic bacteria (g).

### 2.3. Qualitative and Quantitative Evaluation of Chitinase Production from S. cellulosae Actino 48

#### 2.3.1. Detection of Chitinase Production

The promising actinobacterial isolate Actino 48 identified as *S*. *cellulosae*, which produced a higher inhibition percentage against *S*. *rolfsii* than other isolates, was streaked onto a colloidal chitin agar plate and incubated at 30 °C for 10 days. The formation of a halo zone surrounding the colony indicated a positive result for chitinase production.

#### 2.3.2. Chitinase Assay

For the chitinase assay, a fresh culture of *S*. *cellulosae* isolate Actino 48 (10^7^ colony forming units (CFU) mL^−1^) was grown in minimal liquid medium (MLM, containing (g/L) MgSO_4_.7H_2_O, 0.2; K_2_HPO_4_, 0.9; KCl, 0.2; NH_4_NO_3_, 1.0; FeSO_4_.7H_2_O, 0.002; MnSO_4_, 0.002; ZnSO_4_, 0.002; pH 6.8), supplemented with colloidal chitin (1% *w*:*v*) (LOBA Chemie PVT. LTD., Maharashtra, India) and incubated for 8 days at 30 °C in flasks. Samples were used for the colorimetric estimation of chitinase every day using the method of Boller and Mauch [27]. We incubated 1 mL of cell-free supernatant with 1 mL of colloidal chitin (1% *v*:*v*) in a citrate phosphate buffer (0.1 M pH 6.5) at 40 °C for 2 h in a shaking water bath. The reaction was stopped by adding 2 mL 3,5-dinitrosalicylic acid (DNS) reagent and kept in a boiling water bath for 5 min to develop the color. The tubes were cooled, centrifuged at 5000× *g* for 10 min, and absorbance was measured at optical density (OD) 575 nm against the blank prepared with 0.1 M citrate phosphate buffer and 0.45% colloidal chitin without enzyme. One unit of chitinase is defined as the amount of enzyme that releases 1 μmol of N-acetylglucosamine per minute under the reaction condition.

### 2.4. Detection of Interaction between S. cellulosae Actino 48 and S. rolfsii

Scanning electron microscope (SEM, JEOL JSM-6360LA, Tokyo, Japan) was used to detect and analyze the inhibition interaction between *S*. *rolfsii* and *S*. *cellulosae* Actino 48. A dual-culture agar plate assay was used to detect the previous interaction. 

### 2.5. Formulation of Culture Broth, Cell-Free Supernatant, and Cell Pellet Suspension of Chitinase-Producing S. cellulosae Actino 48

Culture broth, cell-free supernatant, and cell pellet suspension of the antagonistic chitinase-producing *S*. *cellulosae* isolate Actino 48, which showed a higher inhibition percentage against *S*. *rolfsii* than other actinobacterial isolates, were used for the preparation of a bioformulation to reduce peanut soil-borne diseases. Talc powder (TP) was used as a carrier for the preparation of biofriendly formulations. We added 10 g of colloidal chitin as a carbon source and an adhesive agent to 400 mL of culture broth (including 10^7^ CFU mL^−1^ of *S*. *cellulosae* Actino 48), cell-free supernatant, and cell pellet suspension. The broth, supernatant, and pellet including additives were mixed homogeneously in a vortex mixer. We adjusted the pH of the formulations to 7.0 by adding 15 g of calcium carbonate to 1 kg of sterilized talc powder (TP) and combined well. We mixed 400 mL of culture broth, cell-free supernatant, and cell pellet suspension with additives with 1 kg of talc powder. The humidity content of bioformulations was decreased to less than 20% by drying, and the bioformulations were stored at 4 °C until use [28].

### 2.6. Characterizations of Talc-Based Bioformulations of Chitinase-Producing S. cellulosae Actino 48

#### 2.6.1. Scanning Electron Microscopy 

The morphological features and microstructure of all talc-based formulations of *S*. *cellulosae* isolate Actino 48 were examined using SEM (JEOL JSM-6360LA, Tokyo, Japan). The sample was operated at an acceleration voltage of 10 KV. Magnification power varied from 300 to 5000×.

#### 2.6.2. Fourier Transform Infrared (FTIR) Spectroscopy

The surface functional groups with binding sites and the structure of the materials used in talc formulations were studied by Fourier transform infrared spectroscopy (FTIR) (Shimadzu FTIR-8400 S, Kyoto, Japan).

#### 2.6.3. Particle Size Analysis

A particle size analyzer (PSA; Mod.: N5, Beckman Coulter, Brea, CA, USA) was used to detect the size of particles of talc-based formulations of *S*. *cellulosae* isolate Actino 48.

### 2.7. Application of Bio-Friendly Formulations of Chitinase-Producing S. cellulosae Actino 48 as Biocontrol Agents against S. rolfsii on Peanut

#### 2.7.1. Preparation of Fungal Inoculum

Sorghum, coarse sand, and water (2:1:2 *v*/*v*) medium was prepared for inoculation of *S*. *rolfsii*. After sterilization, the medium was inoculated using agar discs, obtained from the margin of a 4-day-old colony of the tested fungus. The inoculated media were incubated at 28 °C for 2 weeks and then used for soil infestation [29].

#### 2.7.2. Soil Infestation

Inoculum of *S. rolfsii* was added to the soil surface of each pot at the rate of 2% *w*/*w* and was covered with a thin layer of sterilized soil. The infested pots were irrigated and kept for 14 days before sowing.

#### 2.7.3. Application Dose of Bio-Friendly Formulations and Recommended Fungicide Rizolex-T 50% Wettable Powder (WP)

Seeds of peanut (Giza 6 cv.) were treated with talc-based culture, supernatant, and pellet formulations of chitinase-producing *S*. *cellulosae* Actino 48 as a seed dressing at a rate of 10 g kg^−1^ of seeds or Rizolex-T 50% WP at a ratio of 3 g kg^−1^ of seeds. Formulations were applied again at a rate of 3 kg acre^−1^ two times, 30 and 50 days after seed sowing, as a soil drench.

#### 2.7.4. Greenhouse Experiment

Talc-based formulations of culture broth, cell-free supernatant, and cell pellet suspension of *S*. *cellulosae* Actino 48 were studied as biofungicides for the biocontrol of peanut damping-off, root, and pod rot diseases caused by *S*. *rolfsii* under greenhouse conditions during the 2017 growing season. Pots (50 cm in diameter) containing sterilized, mixed soil of clay and sand (1:1 *v*/*v*) were infested as mentioned before. Nine treatments were performed as follows: (1) *S*. *rolfsii*, (2) untreated control, (3) formulation of culture broth (Cu-F), (4) formulation of supernatant (Su-F), (5) formulation of pellet (PE-F), (6) *S*. *rolfsii* + culture broth formulation, (7) *S*. *rolfsii* + supernatant formulation, (8) *S*. *rolfsii* + pellet formulation, and (9) *S*. *rolfsii* + Rizolex-T 50% WP. Ten peanut seeds (Giza 6 cv.) were treated and sown per each pot. Four replicates (pots) were used for each treatment. Disease assessments were recorded as previously mentioned.

#### 2.7.5. Open-Field Experiment

During the 2018 and 2019 growing seasons, the field experiments were conducted at El-Nobaria, El-Behaira Governorate, Egypt, to study the effect of the talc-based bioformulations of *S*. *cellulosae* Actino 48 in controlling damping-off, root, and pod rot diseases. The fields had a heavy natural infestation with phytopathogenic fungus *S*. *rolfsii*. Peanut seeds (Giza 6 cv.) were sown in the first week of May through the two evaluated seasons with 10 cm spacing between rows. Talc-based formulations of culture broth, cell-free supernatant, and cell pellet suspension of *S*. *cellulosae* Actino 48 and the fungicide Rhizolex-T 50% WP were applied as previously mentioned. Cultural practices and fertilization for the peanut crop were performed as recommended. The experimental unit area was 10.5 m^2^ (1/400 acre). The treatments were applied using a randomized block design with four replicates. Diseases assessments, peanut yield, and dry weight of shoot and root systems were recorded as mentioned before.

#### 2.7.6. Disease Evaluation

Disease was evaluated according to Hussien et al. [29]. The percentage of damping-off (pre- and post-emergence) was estimated 15 and 45 days after sowing using the following formulas:

% Pre-emergence = No. of non-emerged seedlings/No. of sown seeds × 100,

% Post-emergence = No. of dead emerged seedlings/No. of sown seeds × 100,

% Damping-off = pre-emergence % + post-emergence %.

Percentages of plants infected by root rot and surviving healthy plants were estimated after uprooting (120 days from sowing) as follows:

% Root-rot = No. of plants showing root rot/No. of sown seeds × 100,

% Apparently healthy plants = No. of surviving healthy plants/No. of sown seeds × 100.

Plants in individual pots/plots were dug up and inverted based on an optimum maturity index. Pods were threshed, air-dried for 15 days, weighed, and then examined for pod rot incidence. The percentage of pod rot was recorded as:

% Pod rot = No. of rotted pods/No. of total pods × 100,

% Apparently healthy pods = No. of healthy pods/No. of total pods × 100,

% increase in yield = (Dry weight of total pods in treatment − Dry weight of total pods in control)/Dry weight of total pods in treatment × 100.

### 2.8. Quantitative Real-Time PCR Analysis of the Defense-Related Genes

#### 2.8.1. Plant Total RNA Extraction and cDNA Synthesis

Total RNA was extracted from peanut leaves (0.1 g, fresh weight) collected at 72 and 96 h post-inoculation (hpi) with the talc-based culture, supernatant, and pellet formulations of *S*. *cellulosae* Actino 48 using the guanidium isothiocyanate (GITC) extraction method with some modifications [30]. The purity and concentration of extracted RNA were determined using SPECTROstar Nano (BMG Labtech, Ortenberg, Germany), while the integrity was assessed using agarose gel electrophoresis. One microgram of DNase-treated total RNA was used to synthesize cDNA in a reverse-transcription reaction as described previously [31]. The RT-PCR reaction mixture was stored at –20 °C until use.

#### 2.8.2. qRT-PCR Assay and Data Analysis

The transcriptional levels of three peanut defense-related genes (peroxidase (*POD*), pathogenesis-related protein-1 (*PR-1*), and chitinase (*PR-3*)) in all treatments were evaluated using qRT-PCR at 72 and 96 h post-inoculation with the talc-based culture, supernatant, and pellet formulations of *S*. *cellulosae* Actino 48 (Table 1). The *β-actin* gene (Table 1) was used as a reference gene to normalize the transcript expression levels. Each biological sample was run in triplicate reactions on a Rotor-Gene 6000 (QIAGEN, ABI System, Valencia, CA, USA) using SYBR Green PCR Master Mix (Thermo, Waltham, MA, USA) as previously described [32]. The relative expression level of each tested gene was truthfully calculated according to Reference [33].

### 2.9. Statistical Analysis

The relative expression levels were analyzed by one-way analysis of variance (ANOVA) using the CoStat software, and the significant differences were determined according to the least significant difference (LSD). *p* ≤ 0.05 level of probability and standard deviation (±SD) are shown as a column bar. Compared to the healthy control, the relative expression levels higher than 1 demonstrated an increase in gene expression (upregulation), whereas values lower than 1 indicated a decrease in expression levels (downregulation).

## 3. Results

### 3.1. Antagonistic Effect of Actinobacterial Isolates against S. rolfsii

**Figure 1:**
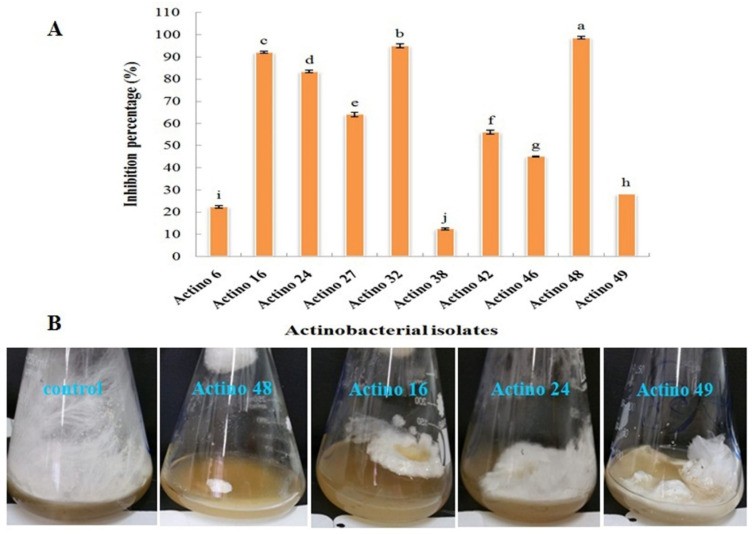
(**A**) Inhibition percentage of actinobacterial isolates against *Sclerotium rolfsii* and (**B**) antagonistic effect of actinobacterial isolates Actino 48, Actino 16, Actino 24, and Actino 49 against *S*. *rolfsii*. Flask on the left in each photo is the corresponding control (fungus) without the antagonist.

Ten actinobacterial isolates were tested as potential biological control agents for their antagonistic effect on the in vitro growth of *S*. *rolfsii*. The data obtained in the current study revealed significant differences between actinobacterial isolates. Isolate Actino 48 was more effective in inhibiting the fungal mycelia growth of *S*. *rolfsii* than other isolates and had the highest inhibition percentage against the pathogen, which reached 98.7%, followed by actinobacterial isolate Actino 32, which reached 95% (Figure 1).

### 3.2. Qualitative and Quantitative of Chitinase Production from S. cellulosae Actino 48

**Figure 2:**
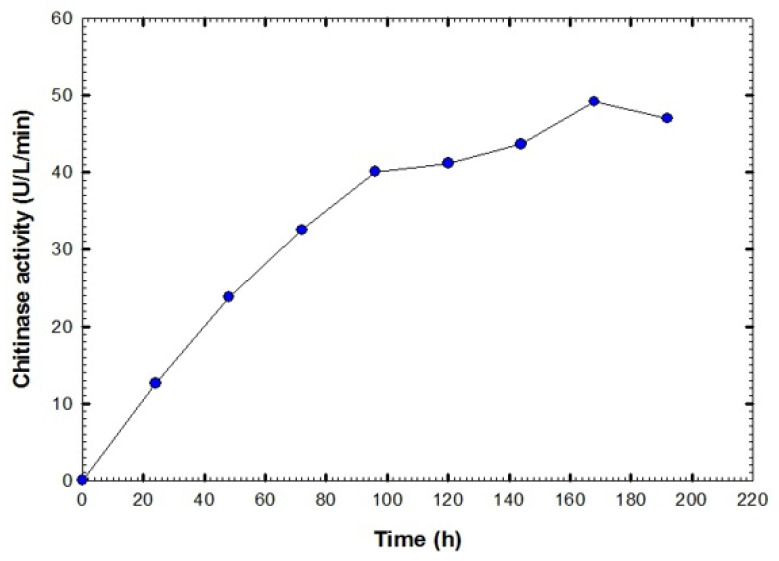
Chitinase activity of cell-free supernatant of *Streptomyces cellulosae* Actino 48.

The ability of the actinobacterial isolate Actino 48, identified as *S*. *cellulosae*, to produce the chitinase enzyme was tested qualitatively on a chitin agar plate. *S*. *cellulosae* Actino 48 formed a large halo zone surrounding the colony, which indicated the chitinase production and chitin degradation abilities. The Actino 48 isolate that showed the highest inhibition percentage against *S*. *rolfsii* was cultured for chitinase production. The maximum chitinase activity was observed at seven days of cultivation (49.2 U L^−1^ min^−1^). After that, enzyme activity decreased to reach 47 U L^−1^ min^−1^ at eight days (Figure 2).

### 3.3. Detection of Interaction between Actinobacteria and S. rolfsii

**Figure 3:**
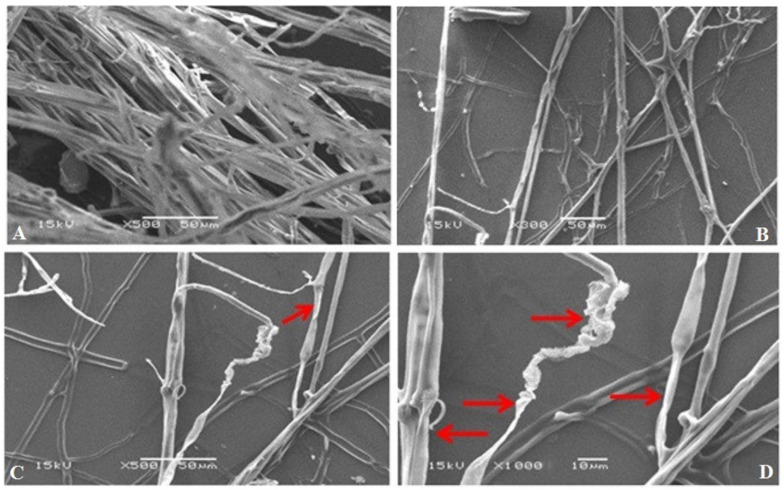
Scanning electron micrographs (SEM) of the antagonistic effect of *Streptomyces cellulosae* Actino 48 against *S. rolfsii*. Micrograph (**A**) is the corresponding fungus control without the antagonistic actinobacterium, and micrographs (**B**), (**C**), and (**D**) are the fungus in the presence of the antagonistic actinobacterium with different magnifications. Arrows indicate abnormality and malformation of the fungal hypha of *S*. *rolfsii*.

Scanning electron microscope (SEM) micrographs of the interaction between *S*. *rolfsii* and chitinase-producing *S*. *cellulosae* Actino 48, which showed a higher inhibition percentage against *S*. *rolfsii* than other isolates, showed abnormal, malformed, and injured fungal hypha of *S*. *rolfsii*, and large losses in the density and mass of the mycelia (Figure 3).

### 3.4. Characterization of Talc-Based Formulations of S. cellulosae Actino 48

#### 3.4.1. SEM

**Figure 4:**
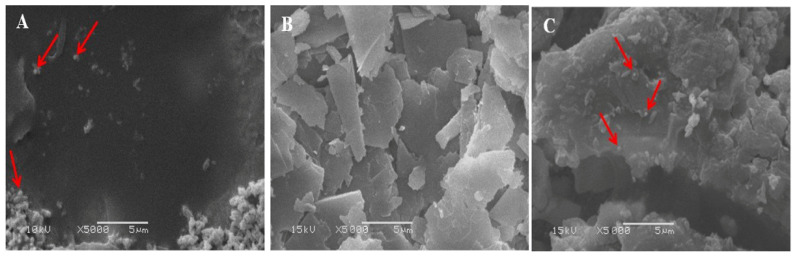
Scanning electron micrographs of talc-based formulations of *Streptomyces cellulosae* Actino 48 (**A**) culture, (**B**) supernatant, and (**C**) pellet formulations. Arrows indicate the presence of *S. cellulosae* Actino 48 spores in bioformulations.

We used SEM for the morphological analysis of talc-based culture broth, cell-free supernatant, and pellet formulations of *S*. *cellulosae* Actino 48 under different magnifications. Figure 4 illustrates talc formulation at 5000×: it has both small and big particle aggregates with sharp edges. The spores of *S*. *cellulosae* Actino 48 are shown on talc particles in the culture broth and pellet formulations.

#### 3.4.2. FTIR Spectroscopy

**Figure 5:**
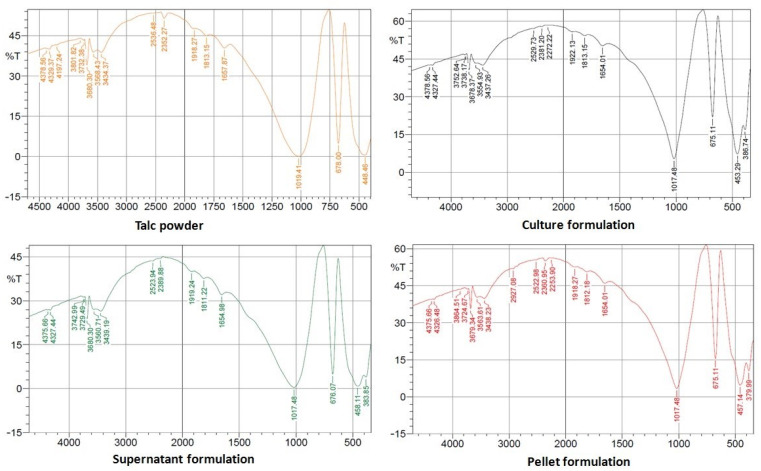
Fourier transform infrared spectroscopy (FTIR) spectrum of talc-based formulations of *Streptomyces cellulosae* Actino 48.

The vibrations in the bands of the FTIR spectrum for talc powder and talc-based culture, supernatant, and pellet formulations of *S*. *cellulosae* Actino 48 are shown in Figure 5, where wave numbers of 3680, 3434, 1658, 1019, 678, and 449 cm^−1^ dominate in the FTIR spectrum of talc powder. The main wave numbers are 3678, 3437, 1654, 1018, 675, and 453 cm^−1^ for the talc-based culture formulation, 3680, 3439, 1655, 1018, 676, and 458 cm^−1^ for the talc-based supernatant formulation, and 3679, 3438, 1654, 1018, 675, and 457 cm^−1^ for the talc-based pellet formulation. The siloxane group (Si–O–Si) stretching vibrational bands were observed with intense peaks at 449 to 458 and 1018 to 1019 cm^−1^, while the bands at 675 to 678 cm^−1^ reflect the Si–O–Mg bond. The peaks located at 1654 to 1685 cm^−1^ are characteristic of the C=C stretching band, representing alkene groups. The bands at 3434 to 3439 and 3678 to 3680 cm^−1^ are assigned to the vibrations of hydroxyl groups linked to Si (Si–OH) and Mg (Mg–OH), respectively.

#### 3.4.3. Particle Size Analysis

**Figure 6:**
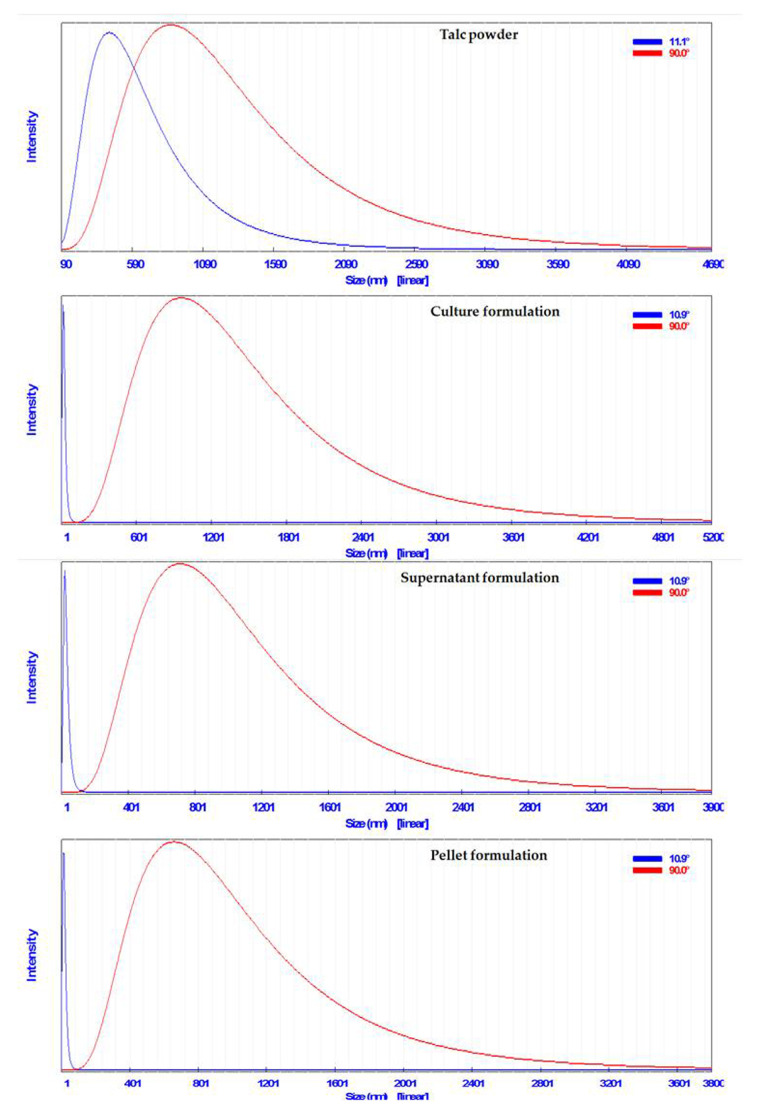
Particles size of talc-based formulations of *Streptomyces cellulosae* Actino 48.

The particles size of talc powder was 958 nm, whereas those of talc-based culture, supernatant, and pellet formulations of *S*. *cellulosae* Actino 48 were 1070, 796, and 754 nm, respectively (Figure 6).

### 3.5. Application of Bio-Friendly Formulations of Chitinase-Producing S. cellulosae Actino 48

#### 3.5.1. Greenhouse Experiment

The talc-based culture broth, cell-free supernatant, and pellet bioformulations of chitinase-producing *S*. *cellulosae* Actino 48 were evaluated to reduce peanut soil-borne diseases caused by *S*. *rolfsii* compared to the recommended fungicide (Rizolex-T) under artificial infection and greenhouse conditions.

**Figure 7:**
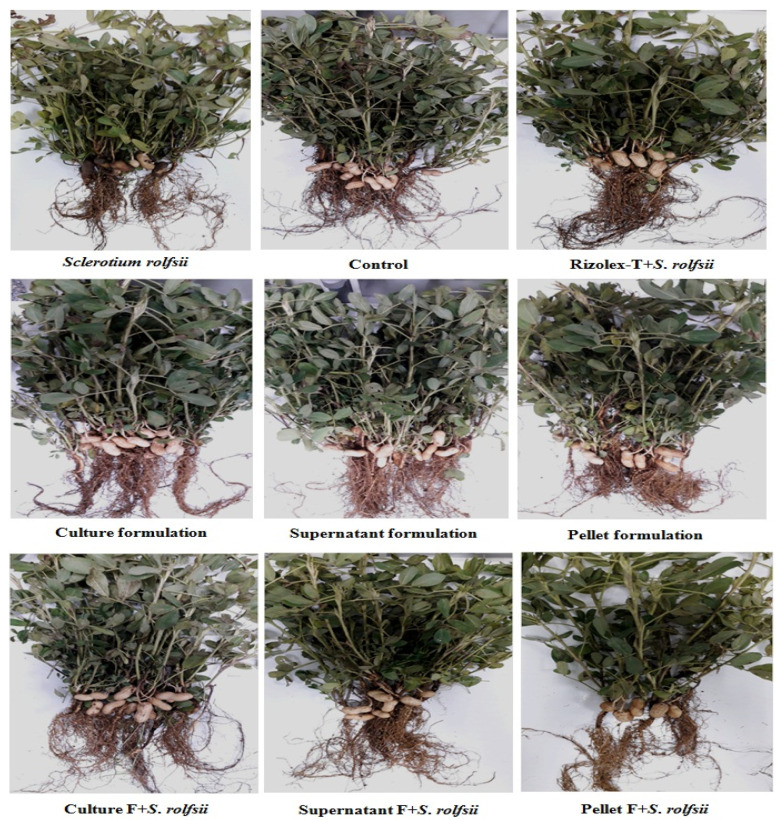
Effect of talc-based culture, supernatant, and pellet formulations of *Streptomyces cellulosae* Actino 48 as biofungicides on peanut root rot and pod rot diseases compared with Rizolex-T as the recommended fungicide under artificial infection and greenhouse conditions.

The data demonstrated in Figure 7 and confirmed in Table 2 show that treatments using talc-based culture and cell-free supernatant formulations of Actino 48 in a soil infected with *S*. *rolfsii* more significantly reduced damping-off percentage, which decreased to 15% and 17.5% respectively, and Rizolex-T reduced damping-off percentage to 12.5% with no significant differences between treatments. Damping-off percentage caused by *S*. *rolfsii* (infected control) was 27.5%. Root rot percentage caused by *S*. *rolfsii* was 32.5%, whereas treatments using talc-based culture and cell-free supernatant formulations effectively reduced root rot percentage, to 12.5% and 20% respectively, with no significant differences between them. Rizolex-T reduced root rot percentage to 10%. The healthy survival percentage of peanut plants for treatments using the talc-based culture formulation of *S*. *cellulosae* Actino 48 and Rizolex-T (standard) in a soil infected with *S*. *rolfsii* increased to 72.5% and 77.5% respectively, and 40% with *S*. *rolfsii* alone. The pellet formulation was less effective than culture and cell-free supernatant formulations in its ability to reduce the incidence of peanut damping-off and root rot diseases.

Mostly all the formulations stimulated the growth of peanut plants whether in uninfected soil or soil infected with *S*. *rolfsii* (Figure 7). Dry weights of shoot and root systems increased significantly in treatments with bioformulations of Actino 48. The talc-based culture formulation more significantly increased the dry weight of shoot and root systems of the peanut in uninfected soil or soil infected with *S*. *rolfsii* than other treatments. The dry weights of the shoot system of peanut plants were 41.56 and 29.70 g, and 4.59 and 3.01 g for the root system of culture formulation in uninfected soil or soil infected with *S*. *rolfsii*, respectively. Treatment with *S*. *rolfsii* alone decreased the dry weights of the shoot and root system to 8.31 and 1.48 g, respectively (Table 3). We found no difference between treatment with the talc-based culture formulation of Actino 48 in soil infected with *S*. *rolfsii* and Rizolex-T treatment on increasing the dry weight of the shoot system of peanut plants, but variations were observed in the root systems (Table 3).

**Figure 8:**
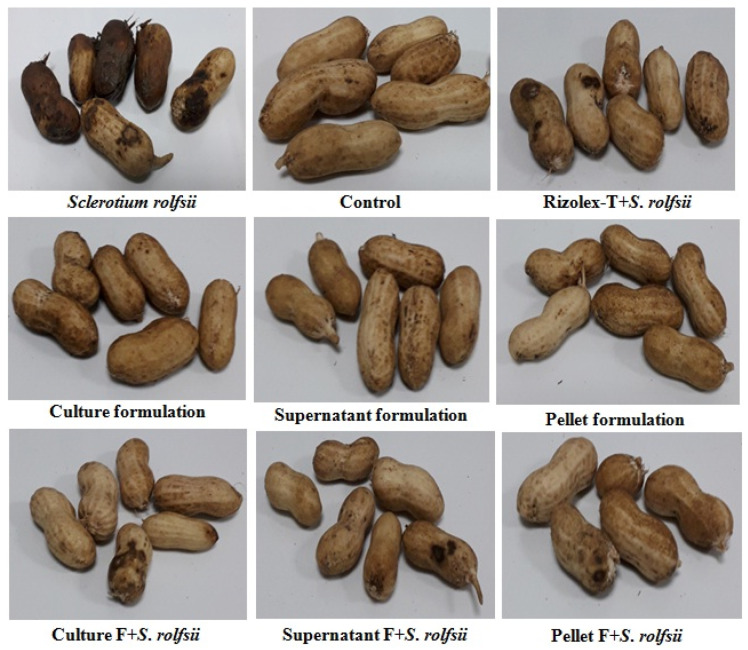
Effect of talc-based culture, supernatant, and pellet formulations of *Streptomyces cellulosae* Actino 48 on peanut pod rot disease compared with Rizolex-T fungicide under artificial infection and greenhouse conditions.

Treatments with talc-based culture, cell-free supernatant, and pellet formulations of Actino 48 in soil infested with *S*. *rolfsii* effectively decreased the percentage of infected peanut pods, which showed no significant differences between them (14.08%, 16.97%, and 16.50%, respectively). The percentage of infected peanut pods in the treatment with *S*. *rolfsii* alone was 56.35% (Figure 8). The same treatments resulted in a high percentage of apparently healthy pods (85.92%, 83.03%, and 83.50%, respectively) compared to the infected treatment, for which we recorded 43.65% healthy pods (Table 3). Treatment with Rizolex-T was highly effective in decreasing the percentage of infected peanut pods, which was 9.13%, for a high percentage of apparently healthy pods of 90.87%.

Table 4 shows the effect of treatment by talc-based culture, cell-free supernatant, and pellet formulations of Actino 48 on peanut yield under greenhouse conditions. Treatments with talc-based culture formulation and Rizolex-T in a soil infested with *S*. *rolfsii* gave high dry weight of healthy pods (11.62 and 10.63 g pot^−1^, respectively) with yield increases of 22.57% and 12.13%, respectively. On the other hand, treatment with *S*. *rolfsii* alone decreased the dry weight of healthy pods to reach 4.59 g pot^−1^ (Figure 8).

#### 3.5.2. Open-Field Experiment

As attained in greenhouse experiments, talc-based culture broth, cell-free supernatant, and pellet bioformulations of chitinase-producing *S*. *cellulosae* Actino 48 were estimated to reduce peanut soil-borne diseases caused by *S*. *rolfsii* compared to the standard fungicide (Rizolex-T) under open-field conditions during two successive seasons, 2018 and 2019. Data presented in Table 5 showed that there were significant effects of all treatments in reducing peanut soil-borne diseases caused by *S*. *rolfsii* during the two tested seasons compared to untreated infected control. In general, Rizolex-T was a more effective treatment in reducing damping-off and root rot diseases caused by *S*. *rolfsii* in the two tested seasons, which reached 8.04% and 7.4% in the first season and 8.26% and 8.7% in the second one respectively, compared to the control (20.22% and 21.52%, and 22.18% and 21.31%). Treatment with talc-based culture broth formulation of chitinase-producing Actino 48 was the most effective one from all bioformulations to reduce the percentage incidence of the two tested diseases, which reached 12.61% and 11.74% in the first season and 13.48% and 10.87% in the second one, respectively. No significant differences, in reducing the percentage incidence of the two tested diseases, showed between treatments of talc-based culture broth and cell-free supernatant in the first season but they showed in the second one. On the other hand, a significant difference was demonstrated between treatments, talc-based culture broth and pellet bioformulations, in the first season but they did not demonstrate to reduce the percentage incidence of the two tested diseases in the second season.

Under open-field conditions and through the first season, 2018, talc-based culture formulation was more effective in increasing the dry weight of shoot and root systems of peanut plants grown in infected soil with *S*. *rolfsii* than other treatments, with dry weight of shoot system reaching 385.01 g and root system reaching 41.65 g for experimental unit followed by Rizolex-T (380.20 g and 40.92 g), without any significant difference compared to untreated and infected control (095.81 g and 19.27 g), as mentioned in Table 6. Moreover, no significant differences were found between treatments, talc-based culture broth bioformulation and Rizolex-T, in the second season, 2019, on dry weight of peanut shoot and root systems grown in infested soil with *S*. *rolfsii*. In addition, no significant differences were found between treatments, cell-free supernatant and pellet bioformulations, in the same season, 2019, but they were less effective than talc-based culture broth bioformulation and Rizolex-T on dry weight of peanut shoot and root systems (Table 6).

As demonstrated in Table 7, competence of talc-based culture broth, cell-free supernatant, and pellet bioformulations of chitinase-producing Actino 48 and Rizolex-T were verified to reduce peanut pods rot incidence (%) under open-field conditions during the two seasons, 2018 and 2019. Rizolex-T and talc-based culture broth gave high percentages of healthy pods (94.26%, 90.93%, and 94.69%, 92.76%) and low percentages of infected pods (5.74%, 9.07%, and 5.31%, 7.24%) in the two seasons respectively, compared to the untreated and infected control. Moreover, no significant differences were found between treatments, cell-free supernatant, and pellet bioformulations in the two tested seasons, 2018 and 2019, but they were less effective than talc-based culture broth bioformulation and Rizolex-T on percentage of infected peanut pods.

Table 8 displays the effect of treatment by different bioformulations of Actino 48 and Rizolex-T on peanut yield under natural infection with *S*. *rolfsii* and open-field conditions. Treatments by Rizolex-T and talc-based culture formulation in a soil infested with *S*. *rolfsii* gave high dry weight of healthy pods (489.94 g and 406.96 g pot^−1^, respectively) with yield increasing by 53.94% and 45.08% respectively, in the first season, 2018, as well as in the second season, 2019. On the other hand, treatment by *S*. *rolfsii* alone decreased the dry weight of healthy pods to reach 194.51 g and 202.14 g pot^−1^ for the two evaluated seasons, respectively.

### 3.6. Quantitative Real-Time PCR Analysis of the Defense-Related Genes

#### 3.6.1. Effects on the Transcriptional Level of Peroxidase (POD)

**Figure 9:**
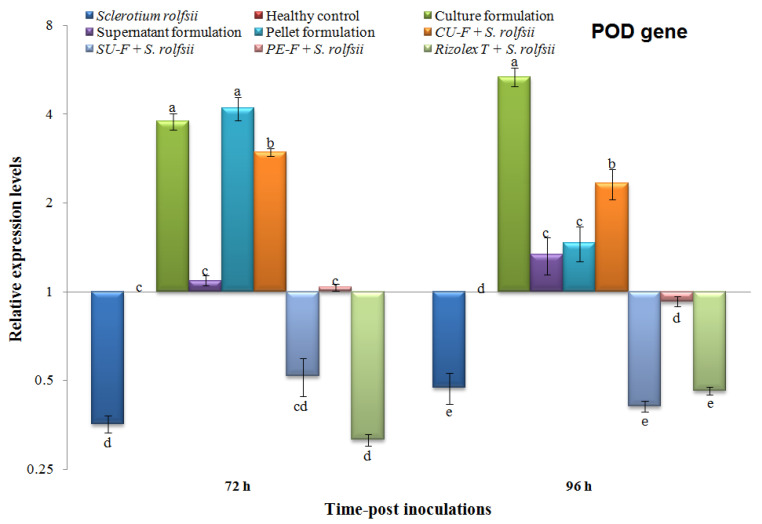
Relative expression levels of peroxidase (*POD*) gene in peanut plants at 72 and 96 h post-inoculation (hpi) of talc-based culture, supernatant, and pellet formulations of *Streptomyces cellulosae* Actino 48 in soil infected with *Sclerotium rolfsii*. Compared to the healthy control, the relative expression levels higher than 1 demonstrate an increase in gene expression (upregulation), while values lower than 1 indicate a decrease in expression levels (downregulation).

Compared to the control, the relative expression levels of POD were differentially expressed at 72 and 92 h in different treatments (Figure 9). The POD transcripts of plants infected with *S*. *rolfsii* were significantly downregulated with relative expression levels 0.356- and 0.473-fold change lower than the control at 72 and 96 h, respectively (Figure 9). Like *S. rolfsii* treatment, the Rizolex-T + *S. rolfsii* treatment exhibited relative expression levels 0.314- and 0.460-fold change lower than control at 72 hand 96 h, respectively. 

Significant upregulation of the POD transcript was observed in plants treated with culture formulation, pellet formulation, and CU-F + *S. rolfsii* at the two-time intervals (Figure 9). The highest expression level was observed in the pellet and culture formulation treatments, with no significant differences, followed by CU-F + *S*. *rolfsii* with relative expression levels 4.119-, 3.784-, and 2.969-fold change increased compared to the control at 72 h, respectively. At 96 h, the highest level (5.329-fold) was demonstrated by culture formulation treatment, followed by the CU-F + *S. rolfsii* treatment with a relative expression level 2.329-fold higher than the control (Figure 9).

#### 3.6.2. Effects on Transcriptional Level of Pathogenesis-Related Protein 1 (PR-1)

**Figure 10:**
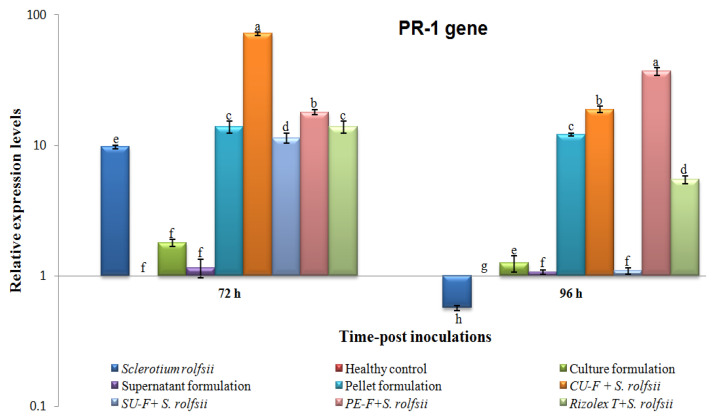
Relative expression levels of *PR-1* gene in peanut plants at 72 and 96 hpi of talc-based culture, supernatant, and pellet formulations of *Streptomyces cellulosae* Actino 48 in a soil infected with *Sclerotium rolfsii*. Compared to the healthy control, the relative expression levels higher than 1 indicate an increase in gene expression (upregulation), while values lower than 1 indicate a decrease in expression levels (downregulation).

Compared with the control at 72 h, the relative expression level of PR-1 was induced in different treatments (Figure 10). The highest transcriptional level (71.671-fold) was shown in the CU-F + *S. rolfsii* treatment, followed by PE-F + *S*. *rolfsii*, which achieved a 17.959-fold higher change than *S*. *rolfsii* treatment, which showed 9.713-fold (Figure 10). Although culture and supernatant formulation treatments showed slight increases of 1.794- and 1.155-fold change respectively, no significant changes were found compared with the control. At 96 h, the transcriptional levels of PR-1 dramatically decreased in all treatments except for the PE-F + *S*. *rolfsii* treatment, in which levels increased and exhibited the highest expression level (37.014-fold) compared with the control (Figure 10). The transcript of PR-1 of *S*. *rolfsii*-treated plants was significantly downregulated with a relative expression level of 0.567-fold lower than the control (Figure 10). PR-1 transcripts with relative expression levels were 18.895-, 12.125-, and 5.464-fold higher than the control in CU-F + *S*. *rolfsii*, pellet formulation, and Rizolex-T + *S*. *rolfsii* at 96 h, respectively (Figure 10).

### 3.7. Effects on Transcriptional Level of Chitinase (PR-3)

**Figure 11:**
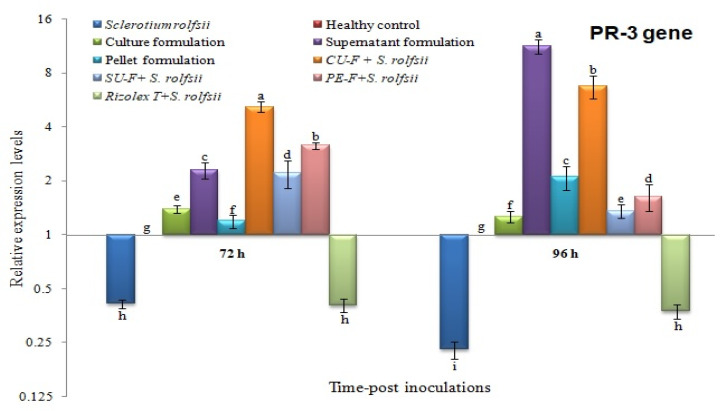
Relative expression levels of *PR*-3 gene in peanut plants at 72 and 96 h post-inoculation (hpi) of talc-based culture, supernatant, and pellet formulations of *Streptomyces cellulosae* Actino 48 in soil infected with *Sclerotium rolfsii*. Compared to the healthy control, the relative expression levels higher than 1 demonstrated an increase in gene expression (upregulation), while values lower than 1 indicated a decrease in expression levels (downregulation).

Similar to POD, peanut plants in both *S*. *rolfsii* and Rizolex-T + *S*. *rolfsii* treatments showed downregulation of PR-3 with relative expression levels of 0.414- and 0.406-fold change lower at 72 h respectively, and 0.229- and 0.376-fold change lower than the control at 96 h, respectively (Figure 11). At 72 h, the highest transcriptional level of PR-3 (5.187-fold) was exhibited in CU-F + *S*. *rolfsii*-treated plants, whereas at 96 h, the supernatant formulation treatment showed the higher expression level, 11.236-fold change higher than healthy control, followed by the CU-F + *S*. *rolfsii* treatment, which was 6.727-fold higher (Figure 11). The moderate induction of PR-3 was observed in the culture formulation, SU-F + *S. rolfsii*, and PE-F + *S*. *rolfsii* at 72 and 96 h with relative expression levels of 1.390-, 2.211-, and 3.149-fold, and 1.263-, 1.357-, and 1.636-fold, respectively (Figure 11).

## 4. Discussion

Given the increasing demand for peanut as food and an oilseeds crop, peanut production must be substantially increased. The national production of comestible oils must be raised to reduce the need to import oils. The huge number of pathogenic fungi affecting the peanut crop is a task for phytopathologists concerned with enhancing peanut yield. Incidences of major fungal diseases can decrease the productivity as much as 50%. Given the changing climatic conditions and reports of incidences of minor diseases becoming virulent, many diseases pose threats to peanut production. Given the health hazards and environmental concerns due to the indiscriminate use of pesticides, biological control agents have been developed. Native bioagents and plant growth-promoting potential are being investigated to control fungal phytopathogens of peanut. Bacteria isolated from the rhizosphere and belonging to a wide variety of genera have the potential to suppress diseases caused by soil-borne phytopathogens [34].

Actinobacteria are considered potential biocontrol agents of plant diseases. Martinez-Alvarez et al. [35] reported that spores-producing bacteria can be used as an alternative to chemical pesticides for controlling plant diseases. Several modes of action of actinobacteria have been suggested as involved in the biocontrol of plant pathogens such as the production of antibiotic compounds, siderophores, hydrogen cyanide (HCN), and hydrolytic enzymes, such as chitinases and glucanases [36,37]. Induced resistance may be implicated in the management of root and pods rot of peanut by actinobacteria. *SAR* and *JA*/*ET* gene expression in *Arabidopsis thaliana* were induced by inoculation with endophytic actinobacteria [38].

In our study, ten actinobacterial isolates were tested in vitro as biocontrol agents for their ability to suppress the mycelium growth of *S*. *rolfsii*. Actinobacterial isolate Actino 48 more effectively inhibited mycelia growth of *S*. *rolfssii* than other actinobacterial isolates. These results agree with those obtained by Adhilakshmi et al. [39], who reported that *Streptomyces* sp. MDU most effectively inhibited the growth of *S*. *rolfsii*. The *B*. *subtilis* isolate B4 showed the strongest antagonistic effect and produced a higher inhibition zone diameter against *S*. *rolfsii* compared to other isolates [34].

Several antibiotics with different chemical structures produced by actinobacteria, such as polyketides, β-lactams, and peptides, show antagonistic effects against bacteria, fungi, and protozoa [40,41]. In addition, various species of *Streptomyces* have the ability to produce chitinase as a lytic enzyme, which works on the chitin of the fungal cell wall, resulting in the suppression of fungal growth [42]. Various chitinolytic *Streptomyces* spp. showed antagonistic activity against *S*. *rolfsii* of chickpea [43]. Ningthoujam et al. [44] reported that chitinase-producing *S*. *vinaceusdrappus* effectively inhibited the mycelial growth of rice fungal pathogens *Curvularia oryzae*, *Pyricularia oryzae*, *Bipolaris oryzae,* and *F*. *oxysporum*. Inhibition of the mycelia growth of *S*. *rolfsii* may be related to the ability of actinobacteria to produce antifungal compounds and lytic enzymes such as chitinase.

Three talc-based bioformulations were prepared using culture broth, cell-free supernatant, and pellet suspension of chitinase-producing *S*. *cellulosae* Actino 48. The bioformulations effectively reduced soil-borne diseases incidence on peanut plants when applied as biocontrol agents in soil infested with *S*. *rolfsii*. Damping-off, root rot, and pod rot and healthy survival percentages were evaluated. In addition, the dry weights of peanut shoot and root systems and the dry weights of infected, healthy, and total pods were determined. 

The obtained data revealed that the talc-based culture broth formulation compared to the talc-based supernatant formulation and talc-based pellet formulation effectively reduced peanut damping-off and root rot diseases caused by *S. rolfsii* under greenhouse and open-field conditions during two successive seasons, 2018 and 2019. Their effects were close to those of Rizolex-T, which is the recommended fungicide. The talc-based culture broth formulation efficiency may refer to this formulation containing *S. cellulosae* Actino 48 spores and chitinase enzyme activity. These results agree with the outcomes reported by Errakhi et al. [45] and Abdel-Gayed et al. [34], who found that *Streptomyces* isolate J-2 and *B*. *subtilis* isolate B4 significantly reduced the disease severity of sugar beet damping-off and root rot of peanut caused by *S*. *rolfsii*, respectively. Zacky and Ting [46] reported that chitinase-producing *Streptomyces* spp. are usually involved in the biocontrol of several plant fungal pathogens and formulated as active biofungicides. A wettable talc powder formulation of *S*. *philanthi* RL-1-178 was more effective in controlling root and stem rot of chili pepper (*Capsicum annuum* L.) caused by *S*. *rolfsii* than granules and encapsulated granules formulations [47].

In this study, a talc-based culture broth formulation of chitinase-producing *S*. *cellulosae* Actino 48 (closed to Rizolex-T) decreased peanut pods rot caused by *S*. *rolfsii* and increased the dry weight of healthy and total pods compared to the control under greenhouse and open-field conditions during two successive seasons, 2018 and 2019. These conclusions agree with those obtained by Abdel-Gayed et al. [34], who reported that a talc-based formulation of *B*. *subtilis* isolate B4 resulted in a higher number of healthy pods and a low percentage of pods infected with *S*. *rolfsii* compared to other treatments under greenhouse and open-field conditions. It also increased the dry weight of healthy and total pods. 

In the current study, we employed qRT-PCR to analyze the expression patterns of some defense genes (*POD*, *PR*-*1*, and *PR-3*) that are regulated in peanut plants in response to *S*. *rolfsii* infection. Talc-based culture, supernatant, and pellet bioformulations of *S*. *cellulosae* Actino 48 were tested against *S*. *rolfsii* under greenhouse conditions. Their effects on the relative expression levels of three defense-related genes (*POD*, *PR-1*, and *PR-3*) at 72 and 96 h post-inoculation were evaluated. The results of our study indicated that treatment with the talc-based culture formulation of *S*. *cellulosae* Actino 48 in soil infected with *S*. *rolfsii* induced higher expression levels of POD, PR-1, and PR-3, which activated the defense mechanism of peanut against *S. rolfsii* infection. Our results agree with those recorded by several investigators who have demonstrated the induction of PR genes and their role in plant disease resistance mechanisms using real-time qPCR. It was reported that the induction of pathogenesis related-protein (PR) genes and increasing POD activity were correlated with activation of the defense system [48,49]. PR-1, a salicylic acid (SA) marker gene, is a principal regulator of systemic acquired resistance (SAR) and could be an indicator of the plant defense response and increasing resistance [50,51,52,53]. Increased POD activity has been associated with improvement in plant defense against pathogens [54,55]. PR-3 (chitinase) is known to inhibit fungal growth and plays an important role in protecting plants from fungal infestations [56]. In the present study, the peanut plants infected with *S. rolfsii* were only associated with downregulation of POD, PR-1, and PR-3 at the two time-intervals, except PR-1 at 72 h showed upregulation with relative expression levels 9.713-fold higher than the control. Compared to plants infected with *S. rolfsii*, the CU-F + *S. rolfsii* plants exhibited the highest transcripts of POD, PR-1, and PR-3 genes with relative 2.969-, 71.671-, and 5.187-fold changes, and 2.329-, 18.895-, and 6.727-fold changes higher than the control at 72 and 96 h, respectively. The changes in the relative expression of studied genes at 72 and 96 hpi may depend on the time of pathogen and/or biocontrol agent treatment. It was reported that the transcriptional levels of many defense-related genes during the plant–fungal interaction depend on the time of the infection process and biocontrol agents treatment [57,58]. As a result, the application of the talc-based culture formulation of *S*. *cellulosae* Actino 48 induced the peanut immune defense system, resulting in the development of SAR activation against *S. rolfsii* infection. These results indicated that *S*. *cellulosae* Actino 48, and specifically the talc-based culture formulation, produces strong biocontrol effects on *S. rolfsii* in peanut and could be used as a biocontrol agent against plant fungal infection. Finally, the physico-chemical structure investigation of a talc-based culture broth formulation of chitinase-producing *S*. *cellulosae* Actino 48 using different techniques such as high-performance liquid chromatography (HPLC) and nuclear magnetic resonance (NMR) could provide an investigation lead for further development of a novel biofungicide. Consequently, we will address these investigations in more details in future studies.

## 5. Conclusions

We can decrease the amounts of chemical fungicides that are broadly applied to control numerous fungal plant diseases using biocontrol agents as an alternative management method. Our study showed that bioformulations of *S*. *cellulosae* Actino 48 can be employed as a biofungicide to control peanut soil-borne diseases caused by *S. rolfsii* as a substitute for chemical fungicides.

## Figures and Tables

**Table 1:** Nucleotide sequences of qRT-PCR primers used in this study.

Primer Name	Abbreviation	Direction	Sequence (5′…3′)
Pathogenesis related protein 1	PR-1	Forward	GTTCCTCCTTGCCACCTTC
		Reverse	TATGCACCCCCAGCATAGTT
Chitinase	PR-3	Forward	ATGGAGCATTGTGCCCTAAC
		Reverse	TCCTACCAACATCACCACCA
Peroxidase	POD	Forward	GGAATGTTGGGTTAGGCAGA
		Reverse	GCTTCCCCTGTTGTGTGAG
Beta-actin	β-actin	Forward	TGGCATACAAAGACAGGACAGCCT
		Reverse	ACTCAATCCCAAGGCCAACAGAGA

**Table 2:** Effect of treatment with talc-based formulations of *Streptomyces cellulosa* Actino 48 on peanut damping-off and root rot incidence (%) under artificial infection and greenhouse conditions.

Treatments	% Damping-Off	% Root Rot	% Survival
*Sclerotium rolfsii*	* 27.5 ± 5.00 ^a^ **	32.5 ± 9.57 ^a^	40.0 ± 14.14 ^f^
Healthy control	07.5 ± 5.00 ^de^	05.0 ± 5.77 ^de^	87.5 ± 05.00^ab^
Culture formulation (CU-F)	05.0 ± 5.77 ^e^	02.5 ± 5.00 ^e^	92.5 ± 09.57 ^a^
Supernatant formulation (SU-F)	07.5 ± 5.00 ^de^	05.0 ± 5.77 ^de^	87.5 ± 05.00 ^ab^
Pellet formulation (PE-F)	07.5 ± 9.57 ^de^	07.5 ± 5.00 ^de^	85.0 ± 12.91 ^abc^
CU-F + *S. rolfsii*	15.0 ± 5.77 ^cd^	12.5 ± 5.00 ^cd^	72.5 ± 09.57 ^cd^
SU-F + S. *rolfsii*	17.5 ± 5.00 ^bc^	20.0 ± 8.16 ^bc^	62.5 ± 12.58 ^de^
PE-F + S. *rolfsii*	25.0 ± 5.77 ^ab^	25.0 ± 5.77 ^ab^	50.0 ± 00.00 ^ef^
Rizolex-T + *S. rolfsii*	12.5 ± 9.57 ^cde^ ***	10.0 ± 8.16 ^de^	77.5 ± 12.58 ^bc^
Least significant difference (LSD 5%)	09.47	09.67	14.64

* All data are averages of four measurements (replicates) ± standard deviation (SD). ** Means in each column followed by the same letter do not differ significantly (*p* ≤ 0.05). *** Significant letters.

**Table 3:** Effect of treatment with talc-based formulations of *Streptomyces cellulosae* Actino 48 on dry weight of shoot and root system, and peanut pods rot incidence (%) under artificial infection and greenhouse conditions.

Treatments	Dry Weight of Shoot System (g pot^−1^)	Dry weight of Root System (g pot^−1^)	Percentage of Infected Pods (%)	Percentage of Apparent Healthy Pods (%)
*Sclerotium rolfsii*	* 08.31 ± 1.47 ^g^ **	1.48 ± 0.28 ^f^	56.35 ± 5.33 ^a^	43.65 ± 5.33 ^e^
Healthy control	36.19 ± 3.23 ^bc^	3.23 ± 0.25 ^bcd^	10.69 ± 5.62 ^de^	89.31 ± 5.62 ^ab^
Culture formulation (CU-F)	41.56 ± 3.79 ^a^	4.59 ± 0.58 ^a^	06.45 ± 0.90 ^e^	93.55 ± 0.90 ^a^
Supernatant formulation (SU-F)	37.94 ± 2.90 ^ab^	3.52 ± 0.42 ^bc^	11.23 ± 5.04 ^cde^	88.77 ± 5.04 ^abc^
Pellet formulation (PE-F)	28.87 ± 2.16 ^e^	3.34 ± 0.42 ^bc^	10.67 ± 3.64 ^de^	89.33 ± 3.64 ^ab^
CU-F + *S. rolfsii*	29.70 ± 3.16 ^de^	3.01 ± 0.12 ^cde^	14.08 ± 2.33 ^bcd^	85.92 ± 2.33 ^bcd^
SU-F+ S. *rolfsii*	26.28 ± 2.54 ^e^	2.80 ± 0.18 ^de^	16.97 ± 1.99 ^b^	83.03 ± 1.99 ^d^
PE-F + S. *rolfsii*	19.95 ± 3.15 ^f^	2.57 ± 0.38 ^e^	16.50 ± 3.72 ^bc^	83.50 ± 3.72 ^cd^
Rizolex-T + *S. rolfsii*	33.17 ± 1.12 ^cd^ ***	3.58 ± 0.41 ^b^	09.13 ± 3.14 ^de^	90.87 ± 3.14 ^ab^
LSD 5%	3.98	0.53	5.57	5.57

* All data are averages of four measurements (replicates) ± standard deviation (SD). ** Means in each column followed by the same letter do not differ significantly (*p* ≤ 0.05). *** Significant letters.

**Table 4:** Effect of treatment with talc-based formulations of *Streptomyces cellulosae* Actino 48 on peanut yield under artificial infection and greenhouse conditions.

Treatments	Dry weight of Infected Pods (g pot^−1^)	Dry Weight of Healthy Pods (g pot^−1^)	Dry Weight of Total Pods (g pot^−1^)
*Sclerotium rolfsii*	* 03.55 ± 1.60 ^a^ **	11.09 ± 6.88 ^c^	14.63 ± 7.92 ^d^
Healthy control	01.74 ± 1.22 ^bc^	24.68 ± 8.62 ^ab^	26.42 ± 7.88 ^abc^
Culture formulation (CU-F)	00.76 ± 0.05 ^c^	30.71 ± 4.71 ^a^	31.48 ± 4.73 ^ab^
Supernatant formulation (SU-F)	01.71 ± 0.43 ^bc^	24.35 ± 8.59 ^ab^	26.06 ± 8.78 ^abc^
Pellet formulation (PE-F)	01.73 ± 0.14 ^bc^	26.26 ± 9.02 ^ab^	27.99 ± 9.06 ^abc^
CU-F + *S. rolfsii*	01.39 ± 0.25 ^bc^	24.18 ± 1.79 ^ab^	25.57 ± 1.96 ^abcd^
SU-F+ S. *rolfsii*	02.27 ± 0.82 ^b^	18.65 ± 1.75 ^bc^	20.92 ± 1.79 ^bcd^
PE-F + S. *rolfsii*	01.68 ± 0.79 ^bc^	16.97 ± 4.83 ^bc^	18.64 ± 5.39 ^cd^
Rizolex-T + *S. rolfsii*	01.09 ± 0.46 ^c^ ***	34.58 ± 13.61 ^a^	35.67 ± 13.58 ^a^
LSD 5%	1.17	10.96	11.10

* All data are averages of four measurements (replicates) ± standard deviation (SD). ** Means in each column followed by the same letter do not differ significantly (*p* ≤ 0.05). *** Significant letters.

**Table 5:** Effect of treatment with talc-based formulations of *Streptomyces cellulosae* Actino 48 on peanut damping-off and root rot diseases incidence (%) under open-field conditions, during 2018 and 2019 seasons.

Treatments	Damping-off (%)	Efficacy (%)	Root Rot (%)	Efficacy (%)	Healthy Survival (%)
Trial field season 2018					
Infected control	* 20.22 ± 1.93 ^a^ **		21.52 ± 9.45 ^a^		58.26 ± 1.88 ^d^
Culture formulation	12.61 ± 1.67 ^c^	37.63	11.74 ± 2.07 ^c^	45.45	75.65 ± 3.69 ^b^
Supernatant formulation	15.43 ± 2.29 ^bc^	23.69	12.83 ± 2.28 ^c^	40.41	71.74 ± 4.52 ^bc^
Pellet formulation	16.74 ± 1.31 ^b^	17.20	16.08 ± 1.12 ^b^	22.22	67.18 ± 0.44 ^c^
Rizolex-T	08.04 ± 1.09 ^d^	60.22	07.40 ± 0.50 ^d^	65.66	84.56 ± 1.09 ^a^
LSD 5%	2.87		2.89		4.64
Trial field season 2019					
Infected control	22.18 ± 1.67 ^a^		21.31 ± 3.06 ^a^		56.51 ± 1.59 ^d^
Culture formulation	13.48 ± 2.30 ^c^	39.22	10.87 ± 1.66 ^cd^	48.98	75.65 ± 2.75 ^b^
Supernatant formulation	16.74 ± 0.83 ^b^	24.51	14.56 ± 1.65 ^b^	31.63	68.70 ± 1.42 ^c^
Pellet formulation	15.87 ± 1.65 ^bc^	28.43	13.26 ± 2.06 ^bc^	37.78	70.87 ± 1.12 ^c^
Rizolex-T	08.26 ± 1.51 ^d^ ***	62.75	08.70 ± 1.23 ^d^	59.18	83.04 ± 2.70 ^a^
LSD 5%	2.67		3.27		3.41

* All data are averages of four measurements (replicates) ± standard deviation (SD). ** Means in each column followed by the same letter do not differ significantly (*p* ≤ 0.05). *** Significant letters.

**Table 6:** Effect of treatment with talc-based formulations of *Streptomyces cellulosae* Actino 48 on dry weight of shoot and root system of peanut under open-field conditions during seasons of 2018 and 2019.

Treatments	Dry weight of shoot system (g plot^−1^)	Increase ^Z^ (%)	Dry weight of root system (g plot^−1^)	Increase ^Z^ (%)
Trial field season 2018				
Infected control	* 095.81 ± 09.37 ^d^ **		19.27 ± 2.75 ^c^	
Culture formulation	385.01 ± 09.90 ^a^	75.11	41.65 ± 1.81 ^a^	53.73
Supernatant formulation	289.05 ± 10.46 ^b^	66.87	32.14 ± 2.20 ^b^	40.03
Pellet formulation	265.91 ± 13.76 ^c^	63.97	34.60 ± 3.55 ^b^	44.30
Rizolex-T	380.20 ± 25.91 ^a^	74.80	40.92 ± 1.44 ^a^	52.87
LSD 5%	21.24		3.94	
Trial field season 2019				
Infected control	091.25 ± 07.96 ^c^		17.81 ± 2.34 ^c^	
Culture formulation	370.47 ± 07.59 ^a^	75.37	39.62 ± 1.61 ^a^	55.06
Supernatant formulation	275.86 ± 22.62 ^b^	66.92	30.80 ± 1.80 ^b^	42.18
Pellet formulation	257.28 ± 15.66 ^b^	64.53	30.96 ± 1.95 ^b^	42.47
Rizolex-T	373.56 ± 25.85 ^a^ ***	75.57	40.28 ± 2.28 ^a^	55.79
LSD 5%	27.89		3.22	

* All data are averages of four measurements (replicates) ± standard deviation (SD). ** Means in each column followed by the same letter do not differ significantly (*p* ≤ 0.05). *** Significant letters. ^Z^ Increase % = [treatment − control]/treatment × 100.

**Table 7:** Effect of treatment with talc-based formulations of *Streptomyces cellulosae* Actino 48 on percentage of infected pods of peanut under open-field conditions during seasons of 2018 and 2019.

Treatments	Percentage of Infected Pods (%)	Percentage of Apparent Healthy Pods (%)
Trial field season 2018		
Infected control	* 47.22 ± 3.47 ^a^ **	52.78 ± 3.47 ^d^
Culture formulation	09.07 ± 1.05 ^c^	90.93 ± 1.05 ^b^
Supernatant formulation	12.43 ± 2.38 ^b^	87.57 ± 2.38 ^c^
Pellet formulation	13.74 ± 1.31 ^b^	86.26 ± 1.31 ^c^
Rizolex-T	05.74 ± 0.75 ^d^	94.26 ± 0.75 ^a^
LSD 5%	3.08	3.08
Trial field season 2019		
Infected control	39.09 ± 1.73 ^a^	60.91 ± 1.73 ^c^
Culture formulation	07.24 ± 0.78 ^c^	92.76 ± 0.78 ^a^
Supernatant formulation	10.61 ± 0.46 ^b^	89.39 ± 0.46 ^b^
Pellet formulation	10.61 ± 1.81 ^b^	89.39 ± 1.81 ^b^
Rizolex-T	05.31 ± 1.02 ^c^ ***	94.69 ± 1.02 ^a^
LSD 5%	2.00	2.00

* All data are averages of four measurements (replicates) ± standard deviation (SD). ** Means in each column followed by the same letter do not differ significantly (*p* ≤ 0.05). *** Significant letters.

**Table 8:** Effect of treatment with talc-based formulations of *Streptomyces cellulosae* Actino 48 on peanut yield under open-field conditions during seasons of 2018 and 2019.

Treatments	Dry Weight of Infected Pods (g plot^−1^)	Dry Weight of Healthy Pods (g plot^−1^)	Dry Weight of Total Pods (g plot^−1^)	Yield Increase ^Z^ (%)
Trial Field Season 2018				
Infected control	* 37.31 ± 4.08 ^a^ **	194.51 ± 09.67 ^d^	231.82 ± 11.85 ^d^	-----
Culture formulation	15.12 ± 1.68 ^c^	406.96 ± 07.15 ^b^	422.08 ± 08.00 ^b^	45.08
Supernatant formulation	19.35 ± 0.76 ^b^	333.81 ± 18.10 ^c^	353.16 ± 17.73 ^c^	34.36
Pellet formulation	21.64 ± 2.09 ^b^	350.37 ± 13.72 ^c^	372.01 ± 14.16 ^c^	37.68
Rizolex-T	13.32 ± 2.35 ^c^	489.94 ± 12.11 ^a^	503.26 ± 13.68 ^a^	53.94
LSD 5%	3.36	20.17	21.58	
Trial Field Season 2019				
Infected control	33.27 ± 3.92 ^a^	202.14 ± 05.17 ^e^	235.41 ± 08.87 ^e^	-----
Culture formulation	13.27 ± 1.52 ^c^	428.55 ± 20.66 ^b^	441.82 ± 21.33 ^b^	46.72
Supernatant formulation	17.08 ± 1.48 ^b^	361.86 ± 13.01 ^c^	378.94 ± 11.59 ^c^	37.88
Pellet formulation	19.17 ± 2.34 ^b^	328.66 ± 05.97 ^d^	347.83 ± 05.01 ^d^	32.32
Rizolex-T	10.98 ± 1.64 ^c^ ***	482.23 ± 16.57 ^a^	493.21 ± 15.53 ^a^	52.27
LSD 5%	3.51	23.31	23.41	

* All data are averages of four measurements (replicates) ± standard deviation (SD). ** Means in each column followed by the same letter do not differ significantly (*p* ≤ 0.05). *** Significant letters. ^Z^ Increase % = [treatment − control]/treatment × 100.
